# The Relationship between the Prognostic Marker LIMA1 in Head and Neck Squamous Cell Carcinoma and Immune Infiltration

**DOI:** 10.1155/2022/1040116

**Published:** 2022-08-31

**Authors:** Hesen Huang, Yu Du, Dean Zhao, Kaiqin Chen

**Affiliations:** ^1^Department of Otolaryngology-Head and Neck Surgery, Xiang'an Hospital of Xiamen University, Xiamen 361100, Fujian, China; ^2^Department of Neurosurgery, Xiang'an Hospital of Xiamen University, Xiamen 361100, Fujian, China

## Abstract

**Background:**

Head and neck squamous cell carcinoma (HNSC) is one of the most common malignancies, and identification of HNSC biomarkers is critical. LIM Domain And Actin Binding 1 (LIMA1) is involved in actin cytoskeleton regulation and dynamics. The role of LIMA1 in HNSC is unclear. This is the first study to investigate the expression of LIMA1 in HNSC patients and its prognostic value, potential biological functions, and impact on the immune system.

**Methods:**

Gene expression and clinicopathological analysis, enrichment analysis, and immune infiltration analysis were all based on data from The Cancer Genome Atlas (TCGA) with additional bioinformatics analysis. Statistical analysis was performed using TIMER and ssGSEA to analyze the immune response to LIMA1 expression in HNSCs. In addition, Gene Expression Omnibus (GEO), Kaplan–Meier(K-M) survival analysis, and data from the Human Protein Atlas (HPA) were used to validate the results.

**Results:**

LIMA1 played a key role as an independent prognostic factor in HNSC patients. GSEA found that LIMA1 is associated with promoting cell adhesion and suppressing immune function. LIMA1 expression was significantly correlated with infiltration of B cells, CD8+ T cells, CD4+ T cells, dendritic cells, and neutrophils and was coexpressed with immune-related genes and immune checkpoints.

**Conclusion:**

The expression of LIMA1 is increased in HNSC, and the high expression of LIMA1 is associated with poor prognosis. LIMA1 may affect tumor development by regulating tumor-infiltrating cells in the tumor microenvironment (TME). LIMA1 may be a potential target for immunotherapy.

## 1. Foreword

Head and neck squamous cell carcinoma (HNSC) is the most common form of head and neck cancer [1]. HNSC is the sixth most prevalent cancer worldwide [2]; annually, the number of new cases exceeds 600,000 cases [3]. HNSCs are malignant tumors of a paranasal sinus, pharynx, larynx, and oral cavity epithelial cells [4]. Due to smoking, alcohol usage, and the human papillomavirus (HPV), the risk of developing cancer is raised. Despite well-known risk factors such as infection, the etiology of HNSC is exceedingly variable and complex, necessitating further study [5]. Currently, the primary focus of HNSC research is on illness prevention, early screening, the identification of target molecules, and the development of new therapeutics. According to our knowledge, HNSC prognostic prediction still has several deficiencies. In order to enhance the diagnosis and prognosis of HNSC, it is essential to screen for biomarkers with high specificity and sensitivity in order to evaluate personalized therapy regimens.

LIMA1 (LIM Domain And Actin Binding 1), sometimes referred to as EPLIN (Epithelial Protein Lost In Neoplasm), was initially identified as an actin-binding protein that is selectively produced in human epithelial cells [6]. A central LIM domain separates two actin-binding domains in the LIMA1 protein [7]. LIMA1 is controlled by the focal adhesion complex [8] and is responsible for the regulation and dynamics of the actin cytoskeleton, with numerous connections at epithelial cell junctions [6, 9]. LIMA1 contributes to epithelial-mesenchymal transition (EMT) and alters cell-cell adhesion complexes, which are necessary for cancer cell migration and invasion, increase metastatic potential, and are required for cell division [10, 11]. Previous studies have connected LIMA1 to EMT, invasiveness, metastasis, treatment, drug resistance, a poor prognosis, and mortality [12]. In recent years, interest in LIMA1 has increased as its roles have expanded beyond those initially found in cell migration and cytoskeletal dynamics to include gene regulation, the cell cycle, Angiogenesis/lymphangiogenesis, and lipid metabolism [13, 14]. Although HNSC is a widely widespread malignancy, its LIMA1 function is still unknown.

In this study, we gathered data from the TCGA database to examine the expression of LIMA1 in human HNSC samples. Using Xiantao Academic Tools (with R, version 3.6.3), we analyzed the relationship between LIMA1 expression and specific clinical markers and prognosis in HNSC patients. We performed Gene Set Enrichment Analysis (GSEA) in conjunction with enhanced Gene Ontology (GO) and Kyoto Encyclopedia of Genes and Genomes (KEGG) analyses to gain a deeper understanding of the biological processes associated with the LIMA1 regulatory network, which may be responsible for HNSC development. Using TIMER and ssGSEA, we analyzed the connection between LIMA1 and tumor-infiltrating immune cells (TIICs). The connection between LIMA1 and HNSC prognosis was investigated using K-M survival analysis, the HPA, and GEO. This is the first study to examine the connection between LIMA1 and HNSC analyses. The literature indicates that appropriate prognostic models can be used to estimate prognosis [15], which can aid cancer patients in making health care decisions. In addition to investigating the independent risk variables for HNSC, this study established a valuable prognostic model based on these findings.

## 2. Materials and Methods

### 2.1. Information from the TCGA Database

Xiantao Academic collected RNA-seq and miRNA-seq data in various tumor TPM and FPKM formats in the TCGA database and cleaned and filtered the data [16]. This study obtained the data of HNSC in the TCGA database (data type: Clinical Supplement)[17], using the Xiantao academic tool (https://www.xian-tao.love/products), analyzed immune cell infiltration, gene expression (workflow type: HTSeq-TPM), and related clinical information, and visualized the data. The study excluded samples with missing or insufficient information on age, TNM stage, OS time, distant and lymph node metastasis, and local invasion. Save RNA-Seq and clinical data for future investigation. Our survey complies with TCGA's publishing rules. The diagnostic potential of LIMA1 expression as a diagnostic marker was investigated using receiver operating characteristic (ROC) curves.

### 2.2. Gene Set Enrichment Analysis

We obtained normalized RNA-Seq data from TCGA and performed GSEA analysis using Xiantao Academic tools. The number of possible permutations is 1000. Through GSEA analysis, the present study uncovered possible biological roles and pathways for groups with high and low LIMA1 expression. In the enrichment results, a *P* value of 0.05 and a normalized enrichment score (NES) of 25% were considered significant.

### 2.3. Analysis of Immune Infiltration

TIMER (https://cistrome.shinyapps.io/timer) is a comprehensive resource for the systematic examination of immune infiltration in various kinds of cancer. The potential link between LIMA1 expression and TIICs was examined using the TIMER database [18]. TIICs in TIMER include CD4+ T cells, dendritic cells, B cells, CD8+ T cells, neutrophils, and macrophages. TIMER applies deconvolution [19] to gene expression profiles to infer the prevalence of TIIC. The graph generated by TIMER illustrates the relationship between immune cell infiltration levels and HNSC cumulative survival, as well as the effect of gene expression levels on tumor purity [20]. By performing ssGSEA analysis using Xiantao academic tools, this study assessed the infiltration levels of 24 immune cells in tumors. The Spearman correlation approach was used to determine the degree of correlation between LIMA1 and the 24 immune cells described above, as well as the examination of immune cell infiltration in groups with high and low LIMA1 expression. Using a heatmap, which is a graph of the association between each pair of immune cell types in a sample, we analyzed the correlations between the 24 categories of immune cells.

### 2.4. Immune Checkpoint Analysis

As immune checkpoint-related transcripts, PDCD1LG2, CD274, HAVCR2, TIGIT, CTLA4, LAG3, PDCD1, and SIGLEC15 were selected, and their expression data were retrieved. We evaluated the expression of immunological checkpoints and the coexpression of LIMA1 with these immune checkpoints. This study applies the Tumor Immune Dysfunction and Exclusion (TIDE) algorithm to expression profiling data [20] to predict probable immune checkpoint blockade responses.

### 2.5. Comprehensive Analysis

KM conducted a survival research (http://kmplot.com/analysis) to examine the relationship between LIMA1 expression and HNSC patient survival time [21]. Input LIMA1 into the database to acquire a KM survival map. Calculate hazard ratios and log-rank*p*-values.

To calculate the 95% CI and HR, we applied multivariate Cox analysis and assessed the impact of LIMA1 expression and other pathological and clinical factors (sex, age, grade, lymph node, distant metastasis, tumor status, stage, etc.) on OS. On the basis of the results of a multivariate Cox proportional hazards analysis, extremely precise nomograms were produced to forecast the total recurrence rates at 1, 3, and 5 years.

Researchers submitted their own research data to GEO (https://www.ncbi.nlm.nih.gov/gds/) to share data with other researchers freely around the world. We downloaded one series GSE6791 [22] from the GEO database, which was found in GPL570 Platforms ([HG-U133_Plus_2] Affymetrix Human Genome U133 Plus 2.0 Array). To assess differences in LIMA1 expression between HNSCs and normal tissues, we constructed boxplots using GEO to validate our results [23].

Immunohistochemical (IHC) staining was collected from the Human Protein Atlas (HPA) database (https://www.proteinatlas.org). In this study, antibody HPA052645 was employed to analyze immunohistochemical images of LIMA1 protein expression between HNSC tissues in order to examine variations in LIMA1 protein expression at the protein level [24]. In addition, HPA offers RNA measurement services.

### 2.6. Statistical Analysis

All statistical analyses were conducted using R (version 3.6.3). Normalization of gene expression data was done using log2 transformation. Using a two-group*t*-test, normal and malignant tissues were compared. For comparisons between more than or equal to three groups, Kruskal-Wallisone-way ANOVA was applied. For all survival studies, Cox proportional hazards models, KM analysis, and log-rank testing were applied. The correlation between the two variables was analyzed using Spearman's test, with a statistically significant *P* value of 0.05. Due to the lack of complete clinical information in the TCGA database and the fact that not all samples contained clinical baseline information, such as age, TNM stage, and treatment result, it was not able to conduct a comprehensive analysis of each clinical category. Therefore, there is a disparity between the total number of samples and the number of samples in various clinical categories in the table in the Results section.

## 3. Results

### 3.1. Survival Results and Variable Evaluation

**Figure 1:**
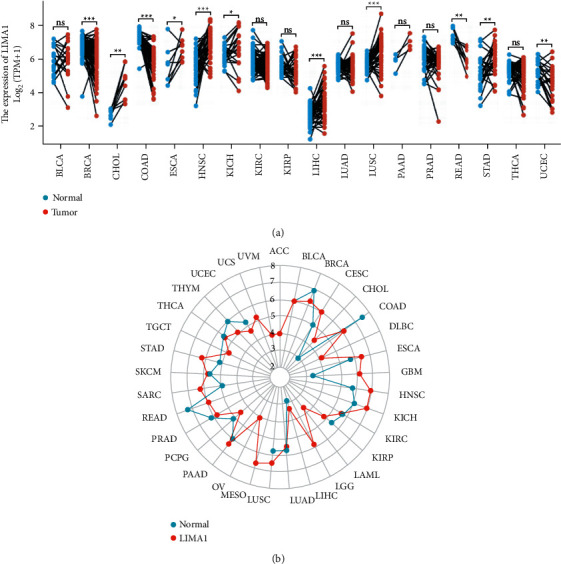
LIMA1 expression in normal and pan-cancer tissues. (a) Paired tissues. (b) Unpaired tissues.

**Figure 2:**
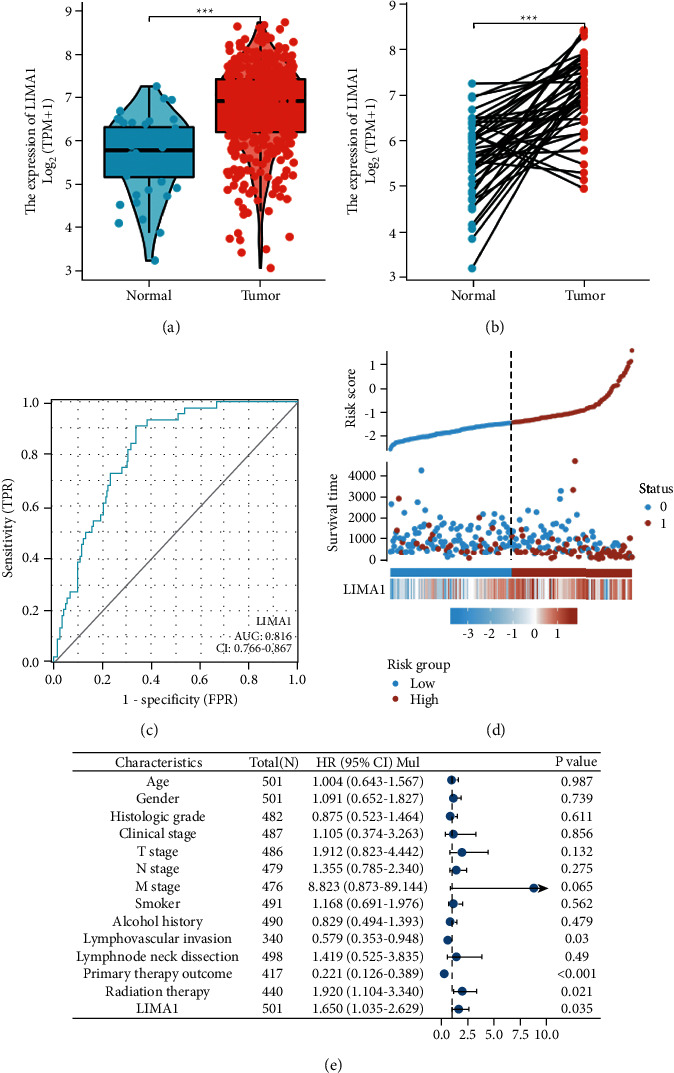
LIMA1 expression in HNSC tissues. (a) LIMA1 expression in normal and unpaired tissues. (b) LIMA1 expression in paired tissues. (c) ROC curve of LIMA1. (d) Risk factors for OS of LIMA1 expression. (e) Forest plot of multivariate Cox analysis of LIMA1 expression and other clinicopathological variables.

Using RNA-seq data from the TCGA database, this study analyzed any differences in LIMA1 mRNA expression between normal and malignant tissues in pan-cancer. Excluding malignancies without normal tissue data, we discovered substantial variations in LIMA1 expression in 11 of 33 cancers, including BRCA, CHOL, COAD, ESCA, HNSC, KICH, LIHC, LUSC, READ, STAD, and UCEC. The expression of LIMA1 was significantly raised in CHOL, ESCA, HNSC, KICH, LIHC, LUSC, and STAD tumors but dramatically decreased in BRCA, COAD, READ, and UCEC tumors (Figure 1). In TCGA, 502 HNSC tumors and 44 normal tissues were further analyzed for unpaired samples, and the expression of LIMA1 in normal and HNSC samples indicated a median difference of 1.111 (0.842–1.379), *P* 0.001 (Figure 2(a)). Compared to matched samples, the difference between the two groups was 1.372% (0.987%–1.7557%), P 0.001 (Figure 2(b)). In the majority of HNSC tumor tissue samples, LIMA1 expression was detected.

### 3.2. Patient Characteristics

**Figure 3:**
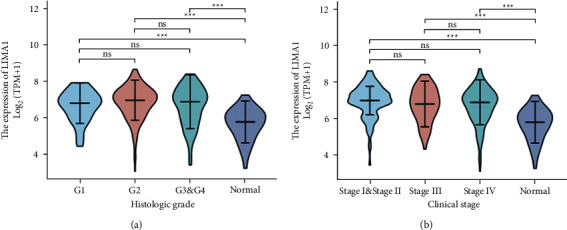
Expression of LIMA1 correlated significantly with histological grade (a), and clinical stage (b).

As indicated in Table 1, the shown baseline data table was acquired from TCGA in May 2022 and includes clinical and gene expression data for 502 primary tumors. The median age of the patients was 61 years, and they were separated into two groups by age: 48.9 percent of patients were under 60 years old, and 51.1 percent of patients were over 60 years old. There were 134 females (26.7%) and 368 males (73.3 percent). 62 cases (12.8 percent) were classified as *G*1, 300 cases (62.1 percent) were classified as *G*2, 119 cases (24.6 percent) were classified as *G*3, and 2 cases (0.4 percent) were classified as *G*4. There were 19 cases (3.9%) of Stage I, 95 cases (19.5%) of Stage II, 102 cases (20.9%) of Stage III, and 272 cases (55.7%) of Stage IV. 33 (6.8 percent) of the 487 patients with *T* stage had *T*1, 144 (29.6 percent) had *T*2, 131 (26.9 percent) had *T*3, and 179 (36.8 percent) had *T*4. There were 239 cases of *N*0 (49.8 percent), 80 cases of *N*1 (16.7 percent), 154 cases of *N*2 (32.1 percent), and 7 cases of *N*3 (1.5 percent). According to *M* staging, *M*0 comprised 472 cases (99%) while *M*1 comprised 5 instances (1 percent). 381 cases (77.4%) were smokers, whereas 333 cases (67.8%) were drinkers. In 122 cases (35.8 percent), the lymphovascular invasion was detected, and lymph node neck dissection was reported in 409 cases (82 percent). In primary outcome therapy, the PD rate was 41 (9.8 percent), the SD rate was 6 (1.4 percent), and the CR rate was 365 (87.3 percent). 287 patients received radiotherapy (65.1%). LIMA1 expression exhibited a promising predictive value, with an AUC of 0.816 on the ROC curve for predicting survival (Figure 2(c)). In addition, the present study demonstrated the distribution of high and low LIMA1 expression in terms of overall survival and risk score (Figure 2(d)). High-risk patients with HNSC exhibited a high level of LIMA1 expression, whereas low-risk patients exhibited a low level of LIMA1 expression. As depicted in Figure 2(e), we employed Cox analysis to investigate the association between LIMA1 expression and OS and other multivariate features of HNSC patients. In conclusion, our multivariate analysis demonstrated that Lymphovascular invasion (HR = 0.579, *p* = 0.03), primary therapy result (HR = 0.221, *p* = 0.001), radiation therapy (HR = 1.920, *p* = 0.021), and LIMA1 (HR = 1.650, *p* = 0.035) were independent risk factors for HNSC.  Figure 3 demonstrates that LIMA1 expression levels were considerably different in HNSC patients with various histologic grades (Figure 3(a)) and clinical stages (Figure 3(b)) relative to the comparable paracancerous tissues.

### 3.3. GSEA Investigation of LIMA1

**Figure 4:**
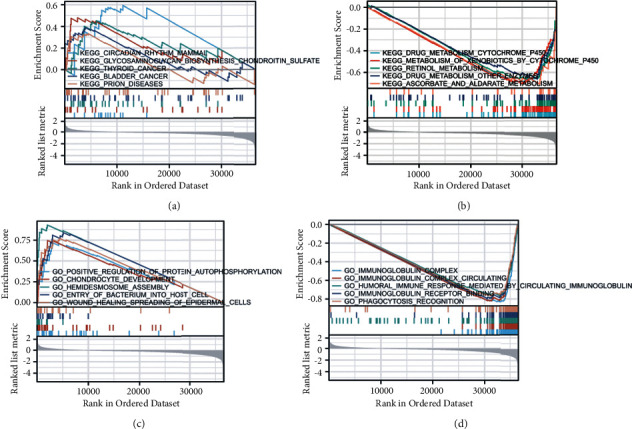
KEGG pathway showed five positively correlated groups (a), and five negatively correlated groups (b). GO term analysis revealed five positively correlated groups (c), and five negatively correlated groups (d).

Analyses of GO terms and KEGG pathways were conducted to investigate the probable biological activities of LIMA1. GSEA demonstrated that the enrichment of KEGG pathways and GO keywords were significantly different (FDR 0.25, *p*-value 0.01) in samples with elevated LIMA1 levels (FDR 0.25, *p*-value 0.04) (Figure 4). We selected the signaling pathways with the highest normalized enrichment values (NES). Five pathways had the strongest positive link with LIMA1 expression, according to KEGG pathway analysis (Figure 4(a)): circadian rhythm animal, glycosaminoglycan biosynthesis chondroitin sulfate, thyroid cancer, bladder cancer, and prion disorders. Drug metabolism of cytochrome, metabolism of xenobiotics by cytochrome P450, retinol metabolism, drug metabolism of other enzymes, and ascorbate and alternate metabolism have the largest negative correlation (Figure 4(b)). As seen in Figure 4(c), GO annotations identified five categories positively connected with high levels of LIMA1: positive control of protein autophosphorylation, chondrocyte development, hemidesmosome assembly, bacterial entry into a host cell, and wound healing epidermal cell spreading. GO analysis identified five categories with the highest negative relationships (Figure 4(d)): immunoglobulin complex, circulating immunoglobulin complex, humoral immune response mediated by circulating immunoglobulin, immunoglobulin receptor binding, and Phagocytosis recognition. These findings show that the emergence and progression of HNSC illness are directly related to LIMA1's functional roles as described below. LIMA1 expression not only promotes the glycosaminoglycan biosynthesis chondroitin sulfate pathway, positive regulation of protein autophosphorylation, and hemidesmosome assembly, but it also inhibits immunoglobulin receptor binding, immunoglobulin-related cycling, and immune-related responses, and it inhibits the metabolism of drugs and other enzymes and cytochromes to exogenous organisms and inhibits the metabolism of endogenous organisms including retinol GO keywords and KEGG pathway analysis demonstrated that LIMA1 enhanced the development of thyroid cancer, bladder cancer, prion disease, and bacterial infiltration into host cells in HNSC.

### 3.4. The Relationship between LIMA1 Expression and TIIC

**Figure 5:**
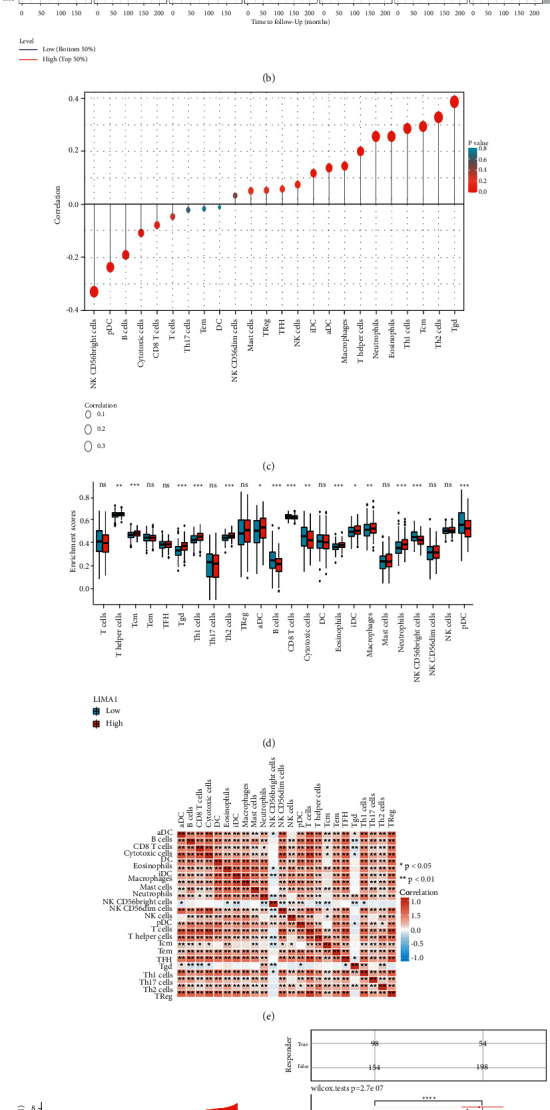
The correlation between LIMA1 expression and immune cell infiltration in tumor microenvironment and the expression of immune checkpoints in HNSC. (a) The relationship between LIMA1 expression and the infiltration level of six types of immune cells. (b) The relationship between immune cell infiltration level and correlation of cumulative survival of HNSCs. (c) Relationship between LIMA1 expression and 24 immune cell types. (d) Change ratio of 24 immune cell subtypes in high and low LIMA1 expression groups in tumor samples. (e) Tumor samples heat map of 24 immune-infiltrating cells in. (f) Differential expression of immune checkpoints with low and high expression of LIMA1. (g) Differences of immune checkpoint blockade with low expression (*G*1) and high expression (*G*2) of LIMA1 reaction.

The immune system is a complex and constantly changing physiological system that functions as a network of connections built by numerous immune cells and immune molecules. At the same time, the immune system, as a defense system, is inseparable from the occurrence and development of cancer [25]. Independent tumor-infiltrating lymphocytes play an essential role in determining overall survival and the status of sentinel lymph nodes [26]. Using TIMER, we investigated the potential relationship between LIMA1 expression and the degree of immune infiltration in HNSCs. LIMA1 expression was substantially linked with B cells (*r* = −0.189, *p*-value = 3.49*e* − 05), CD8 + T cells (*r* = −0.11, *p*-value = 1.67*e* − 02), CD4 + T cells (*r* = −0.11, *p*-value = 1.67*e* − 02), CD4 + T cells (*r* = −0.11, *p*-value = 1.67*e* − 02), and cells (*r* = 0.237, *p*-value = 1). A significant correlation exists. We discovered that HNSC patients with high levels of B cell infiltration (*p*-value = 0.045) had greater cumulative survival, whereas the infiltration levels of the other five immune cell subtypes did not correlate significantly with cumulative survival. Moreover, the study confirmed that HNSC patients with elevated LIMA1 expression had a poor cumulative survival rate (Figure 5(b)). These findings imply that LIMA1 expression levels are related to a worse prognosis and immune infiltration in HNSC patients. LIMA1 plays a crucial function in HNSC immune infiltration.

Next, the link between LIMA1 expression and twenty-four distinct types of immune cells was evaluated in HNSCs. LIMA1 expression is linked positively with iDC, aDC, macrophages, *T* helper cells, neutrophils, eosinophils, Th1 cells, Tcm, Th2 cells, and Tgd and negatively with NK CD56bright cells, pDC, B cells, and Cytotoxic cells (Figure 5(c)). LIMA1 expression levels varied significantly across invading immune cells, including *T* helper cells, Tcm, Tgd, Th1 cells, Th2 cells, aDC, B cells, CD8 T cells, cytotoxic cells, eosinophils, iDCs, macrophages, neutrophils, NK CD56bright cells, and pDC, according to additional research. *T* helper cells, Tcm, Tgd, Th1 cells, Th2 cells, aDC, eosinophils, iDC, macrophages, and neutrophils increased in the LIMA1 high expression group relative to the LIMA1 low expression group, and the changes were statistically significant. There was no statistically significant difference between TFH, Treg, Mast, NK CD56dim, and NK cells (*P* > 0.05). B cells, CD8+ T cells, Cytotoxic cells, NK CD56bright cells, and pDC were reduced in the LIMA1 high expression group compared to the LIMA1 low expression group, and the differences were statistically significant (P 0.05); however, the difference between T cells, Tem, Th17 cells, and DC was not statistically significant (*P* > 0.05) (Figure 5(d)). Additionally, we evaluated potential connections between 24 immune cells (Figure 5(e)). The generated heatmap revealed that the ratios of several subsets of TIIC were moderate to strongly associated.

The expression of immunological checkpoints such as TIGIT, SIGLEC15, CTLA4, HAVCR2, LAG3, CD274, PDCD1, and PDCD1LG2 was studied further in the LIMA1 low expression and high expression groups of HNSCs. Three immunological checkpoints, PDCD1LG2, HAVCR2, and CD274, were considerably upregulated in the LIMA1 high-expression group of HNSCs relative to the LIMA1 low-expression group (Figure 5(f)). In addition, we found that patients with high LIMA1 expression in HNSCs had higher TIDE scores, a poorer response to immune checkpoint blockade (ICB), and shorter survival after receiving ICB than patients with low LIMA1 expression (Figure 5(g)).

### 3.5. Data Verification

**Figure 6:**
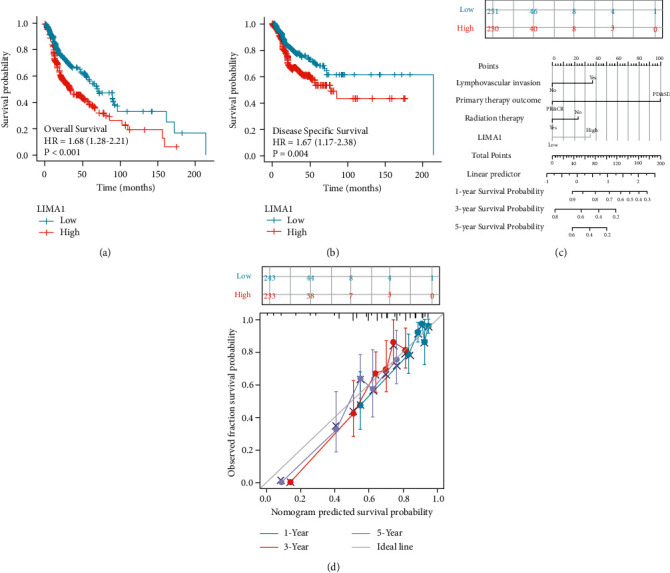
Prognostic analysis of LIMA1 expression. Compared with patients with low LIMA1 expression, patients with high LIMA1 expression had poorer prognosis, including (a) overall survival (OS), (b) disease-specific survival (DSS)) (two log-rank*P* < 0.05). (c) Calibration plot showing the predictive performance of the model constructed using multivariate Cox regression analysis. (d) Multivariate analysis nomogram of clinical features based on LIMA1 expression.

**Figure 7:**
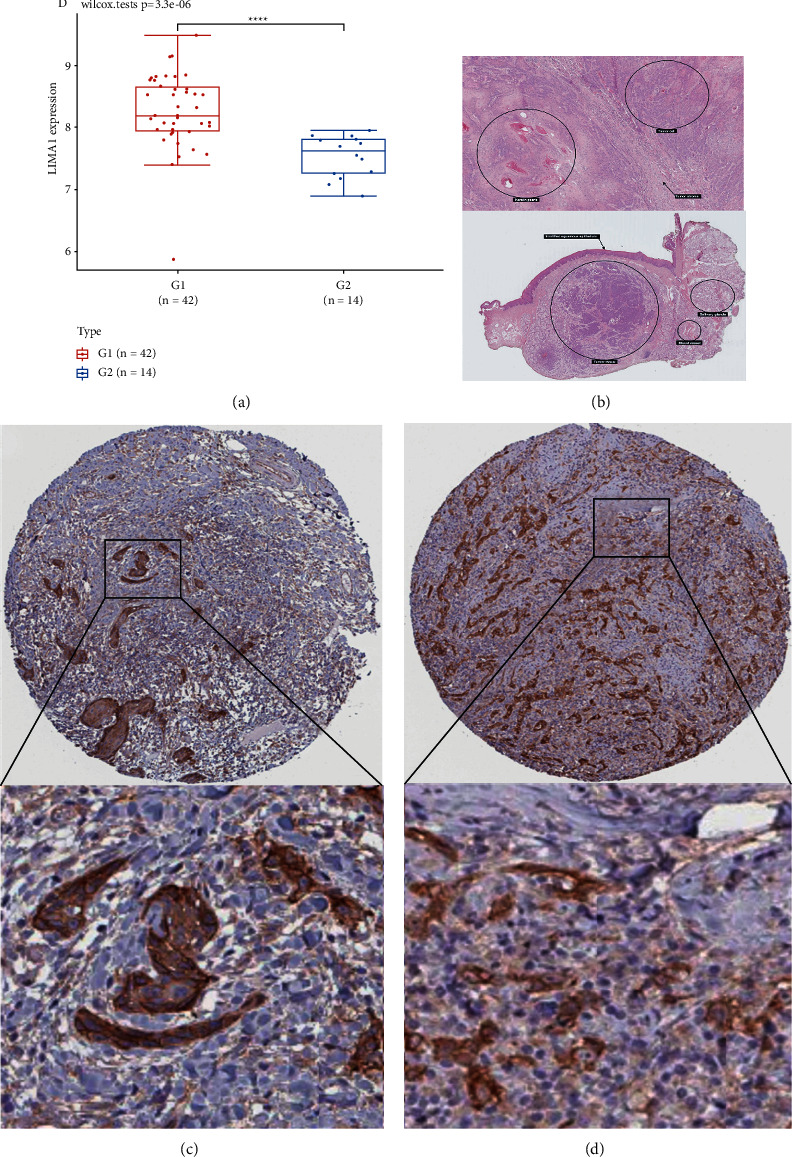
Expression levels of LIMA1 mRNA in normal and HNSC tissues obtained from GEO (a). Hematoxylin-eosin staining of HNSC (b). Observation of HNSC using HPA immunohistochemistry LIMA1 expression in tumor tissue, including (c) LIMA1 medium expression, (d) LIMA1 high expression.

The KM survival plot revealed that the group with high LIMA1 expression had shorter OS (*p*-value 0.001, Figure 6(a)) and DSS (*P*-value = 0.004, Figure 6(b)). Based on the association with HNSC prognosis independent of risk factors, we plotted the nomogram to estimate OS of the disease (Figure 6(c)), and the calibration curve showed good predictive value (Figure 6(d)). Using the GEO database, we determined that LIMA1 mRNA expression was considerably elevated in HNSC compared to the normal group (*p*-value = 3.3*e* − 06, Figure 7(a)). In addition, LIMA1-expressing HNSC tumor tissue was obtained from HPA, and, with this tissue, we were able to: compared to HNSC tumor tissues with low LIMA1 expression, HNSC tumor tissues with high LIMA1 expression stained more intensely (Figures 7(c) and 7(d)).

## 4. Discussion

The majority of HNSC patients are identified at advanced local stages III and IV [27]. Clinically, the most prevalent treatment options for HNSC include surgical resection and adjuvant radiation or concurrent radiotherapy and chemotherapy [28, 29]. Currently, there are no accurate biomarkers for HNSC therapeutic regimens, and the prognosis prediction of these biomarkers is still somewhat controversial [30]. The establishment of distinct prognostic markers for HNSC is therefore helpful for its clinical application [31]. In recent years, biomarkers engaged in multiple linkages of the cell cycle [15, 31–34] have been utilized in the diagnosis of HNSC.

Changes in cell metabolism, growth, adhesion, and proliferation are among the stages of cancer cell development [35]. Cell migration is a complex, closely regulated process involving coordinated changes in signaling, membrane trafficking, and cytoskeletal dynamics. During EMT during development or carcinogenesis, cells undergo extensive genetic and posttranslational programming modifications to enhance biological pathways needed for migration, such as cell-cell adhesion loss, actin polymerization, enhanced protein cytoskeleton, and focal adhesion dynamics regionally [36]. One of the most significant findings regarding LIMA1 is that it regulates actin dynamics by colocalizing with actin filaments and other actin structural regulators and by cross-linking actin filaments, inhibiting branching nucleation via Arp2/3, and influencing cell motility and migration to promote cancer progression [11]. Changes in LIMA1 expression can therefore affect carcinogenesis and cancer progression. However, there are currently no relevant LIMA1 in HNSC research publications.

In the current study, the KEGG pathway and GO term analysis demonstrated that upregulated LIMA1 primarily promotes the essential glycosaminoglycan biosynthesis chondroitin sulfate pathway, positive regulation of protein autophosphorylation, and hemidesmosome assembly function in HNSC and is associated with inhibitory immunoglobulin receptor binding, immunoglobulin-related circulation, and immune-related responses. Concurrently, LIMA1 inhibits the metabolism of medicines and other exogenous enzymes and cytochromes, as well as the metabolism of endogenous organisms such as retinol, ascorbic acid, and aldonic acid. Enhanced LIMA1 expression in HNSCs also increases thyroid cancer, bladder cancer, the progression of prion disease, and bacterial penetration into host cells. We conclude that the overexpression of LIMA1 in HNSC patients promotes cell adhesion and inhibits immune function-related effects, leading to HNSC proliferation, migration, and metastasis. This requires additional research to determine the processes and pathways by which LIMA1 works in HNSCs. Our findings contribute to a better understanding of the biology and function of LIMA1 that makes its overexpression in HNSCs so detrimental. There is no research and application of LIMA1 inhibitors for HNSC at this time. Future development and utilization of LIMA1 inhibitors in HNSC will be facilitated by our research findings.

The immunological microenvironment is critical to the development and incidence of tumors [37,38]. Not only can TME profiles serve as biomarkers to evaluate tumor cell response to immunotherapy, but they can also affect the prognosis of tumors [39]. Numerous investigations have demonstrated that the microenvironment of HNSC tumors contributes to immunological alterations during HNSC progression [40–42], a process involving numerous known and unknown pathways. By evaluating a single-cell database, our study is the first to identify LIMA1 as an essential immune-related prognostic marker in the HNSC tumor microenvironment. Using the TIMER database, we discovered a correlation between LIMA1 expression and the degree of immune infiltration in HNSCs. LIMA1 was strongly negatively connected with B Cells and CD8+ *T* Cells, as shown in Figure 5(a), and considerably favorably correlated with CD4+ T cells, neutrophils, and dendritic cells. The cumulative survival rate of HNSC patients with significant levels of B-cell infiltration was also shown to be greater in Figure 5(b). The current investigation explored the variations in immune cell infiltration between HNSC patients with high and low LIMA1 expression in Figure 5(c). These associations show that LIMA1 plays a critical role in modulating HNSC tumor immunology. In Figure 5(d), the results demonstrated statistically significant differences in the LIMA1 expression levels of immune cells. Collectively, our results imply that LIMA1 plays a crucial role in regulating the immunological activity of HNSCs. To comprehend the link between LIMA1 and immune cells in vivo with greater precision, however, controlled studies and multicenter clinical trials must be conducted.

In recent years, tumor immunotherapy has attracted more and more attention and research. Tumor immunotherapy is beneficial to improve overall survival and long-term therapeutic benefit without significant side effects. By modulating/promoting the patient's immune system to kill cancer cells, various immunotherapeutic approaches have emerged as promising antitumor modalities, especially those that unleash anticancer immunity through immune checkpoint inhibitors [43]. This study found that LIMA1 was coexpressed with immunological checkpoints such as PDCD1LG2, HAVCR2, and CD274. Moreover, patients with high LIMA1 expression demonstrated greater expression at these immunological checkpoints than patients with low LIMA1 expression. Patients with high LIMA1 expression in HNSC responded less favorably to immune checkpoint blockade therapy than those with low LIMA1 expression, resulting in reduced efficacy. Patients with higher LIMA1 expression in HNSC had a shorter survival time following treatment with immune checkpoint blocking (ICB). Based on the results of the GO enrichment analysis, we considered the potential cause for this result, as patients with high LIMA1 expression in HNSCs exhibited greater inhibition of immunoglobulin receptor binding, immunoglobulin-related circulation, and immune-related responses than those with low LIMA1 expression. As a result, we believe that LIMA1 modulates immunological function and immunity to play a significant role in the start and progression of HNSC.

Nevertheless, our study has several drawbacks. First, this study's data are taken from regularly updated and expanded online platform databases; hence, the study's findings are susceptible to change. Second, our study lacked information regarding difficulties and in-depth therapy options. Thirdly, in vivo and in vitro studies were not undertaken to confirm the role of LIMA1 in HNSC immunity and its molecular mechanism. In future studies, we will pay greater attention to the total baseline information of patients and conduct further tests to corroborate the anticipated results.

This is the first study to identify LIMA1 as a novel prognostic marker in HNSC and to link it to immune function. With a clearer understanding of its functional breadth, LIMA1 could serve as a useful diagnostic and therapeutic tool for HNSC, and biomarker therapy could represent a viable future treatment option for HNSC. The mechanism through which LIMA1 promotes tumor growth and metastasis in HNSCs warrants additional study.

## Figures and Tables

**Table 1:** Baseline data on LIMA1 expression in HNSC patients.

Characteristic	Levels	Overall
*n*		502
Age, median (IQR)		61 (53, 69)

Age, *n* (%)	≤60	245 (48.9%)
	>60	256 (51.1%)

Gender, *n* (%)	Female	134 (26.7%)
	Male	368 (73.3%)

Histologic grade, *n* (%)	*G*1	62 (12.8%)
	*G*2	300 (62.1%)
	*G*3	119 (24.6%)
	*G*4	2 (0.4%)

Clinical stage, *n* (%)	Stage I	19 (3.9%)
	Stage II	95 (19.5%)
	Stage III	102 (20.9%)
	Stage IV	272 (55.7%)

*T* stage, *n* (%)	*T*1	33 (6.8%)
	*T*2	144 (29.6%)
	*T*3	131 (26.9%)
	*T*4	179 (36.8%)

*M* stage, *n* (%)	*M*0	472 (99%)
	*M*1	5 (1%)

*n*		502

*N* stage, *n* (%)	*N*0	239 (49.8%)
	*N*1	80 (16.7%)
	*N*2	154 (32.1%)
	*N*3	7 (1.5%)

Smoker, *n* (%)	No	111 (22.6%)
	Yes	381 (77.4%)

Alcohol history, *n* (%)	No	158 (32.2%)
	Yes	333 (67.8%)

Lymphovascular invasion, *n* (%)	No	219 (64.2%)
	Yes	122 (35.8%)

Lymph node neck dissection, *n* (%)	No	90 (18%)
	Yes	409 (82%)

Primary therapy outcome, *n* (%)	PD	41 (9.8%)
	SD	6 (1.4%)
	PR	6 (1.4%)
	CR	365 (87.3%)

Radiation therapy, *n* (%)	No	154 (34.9%)
	Yes	287 (65.1%)

## Data Availability

The data used to support the findings of this study are included within the article, and the datasets and code can be obtained from the corresponding author upon reasonable request.
